# A Medal in the Olympics Runs in the Family: A Cohort Study of Performance Heritability in the Games History

**DOI:** 10.3389/fphys.2018.01313

**Published:** 2018-09-18

**Authors:** Juliana Antero, Guillaume Saulière, Adrien Marck, Jean-François Toussaint

**Affiliations:** ^1^Institut de Recherche bioMédicale et d’Epidémiologie du Sport, Institut National du Sport de l’Expertise et de la Performance, Paris, France; ^2^EA 7329, Université Paris Descartes, Sorbonne Paris Cité University, Paris, France; ^3^CIMS, Hôtel-Dieu, Assistance Publique, Hopitaux De Paris, Paris, France

**Keywords:** performance, heritability, athletes, family twin study, genetics

## Abstract

**Introduction:** Elite performance in sports is known to be influenced by heritable components, but the magnitude of such an influence has never been quantified.

**Hypothesis/Objectives:** We hypothesized that having a former world-class champion in the family increases the chances of an athlete to repeat the achievement of her or his kinship. We aimed to measure the heritability of a medal in the Olympic Games (OG) among Olympians and to estimate the percentage of the genetic contribution to such a heritance.

**Study Design:** Twin-family study of a retrospective cohort.

**Methods:** All the 125,051 worldwide athletes that have participated in the OG between 1896 and 2012 were included. The expected probability to win a medal in the OG was defined as the frequency of medallists among Olympians without any blood kinship in the OG. This expected probability was compared with the probability to win a medal for Olympians having a kinship (grandparent, aunt/uncle, parent, or siblings) with a former Olympian that was a (1) non-medallist or (2) medallist. The heritability of the genetically determined phenotype (h^2^) was assessed by probandwise concordance rates among dizygotic (DZ) and monozygotic (MZ) twins (*n* = 90).

**Results:** The expected probability to win a medal in the OG was 20.4%. No significant difference of medal probability was found in the subgroups of Olympians with a Non-medallist kinship, except among siblings for whom this probability was lower: 13.3% (95% CI 11.2–14.8). The medal probability was significantly greater among Olympians having a kinship with a former Olympic Medallist: 44.4% for niece/nephew (33.7–54.2); 43.4% for offspring (37.4–48.6); 64.8% for siblings (61.2–68.8); 75.5% for DZ twins (63.3–86.6); and 85.7% for MZ twins (63.6–96.9); with significantly greater concordance between MZ than DZ (*p* = 0.01) and h^2^ estimated at 20.5%.

**Conclusion:** Having a kinship with a former Olympic medallist is associated with a greater probability for an Olympian to also become a medallist, the closer an athlete is genetically to such kinship the greater this probability. Once in the OG, the genetic contribution to win a medal is estimated to be 20.5%.

## Introduction

The genetic influence on sports performance is related to the athletes’ intrinsic aspects such as, health, biometric, physiological and psychological factors, predisposition to sustain effort, response to training and recovery/resilience (Pérusse et al., 2013). The identification of multiple genetic polymorphisms favoring athletic skills suggests a strong interdependence influencing elite performance (Bray et al., 2009; Guilherme et al., 2018). A polymorphism in the ACTN3 gene, for instance, has been shown to be associated with muscular performance (Yang et al., 2003, 2009; Papadimitriou et al., 2008; Eynon et al., 2009), while greater frequency of polymorphism in genes associated with sustained effort, such as the ACE or the COL5A1 gene, has been found (Montgomery et al., 1998; Brown et al., 2011; Ma et al., 2013).

These inter-individual variations determine a predisposition to a particular type of effort as well as the athletic level, since the frequency of polymorphisms favoring specific performance increases with the sports level (Eynon et al., 2009, 2012). For instance, previous studies have shown that heterozygous mutation of the HFE gene, which improves oxygen transport, was doubled in endurance athletes compared to the general population and was 12 times more frequent among those reaching international podiums (Hermine et al., 2015). Yet, the genetic contribution to elite athletic status is still barely understood (Pitsiladis et al., 2013; Wang et al., 2013).

In addition, the environment, defined as all the extrinsic aspects surrounding the athletes, contributes to modulate the genomic expression, including psychosocial backgrounds (Côté, 1999), access to training facilities (Ericsson et al., 1993; Baker and Horton, 2004), birthplace (Côté et al., 2006), and family support (Hopwood et al., 2015). Such extrinsic factors promote or limit the transcription of certain genotypes (Davids and Baker, 2012) and are also partially heritable (Stubbe et al., 2005).

Hence an important heritance component influences elite performance (Ostrander et al., 2009). Major selective competitions reinforce this mechanism, reserving access to athletes with rare profiles (Antero-Jacquemin et al., 2015). Then, an individual, in direct relation with a former elite champion, may inherit intrinsic and extrinsic features favoring athletic skills. This heritance probably increases with the degree of relatedness, a measurable indicator of a multifactorial trait, such as performance (Borecki and Province, 2008). For instance, siblings share a higher percentage of genes, as well as environmental factors, compared to grandchildren with their grandparents. Accordingly, dissimilarity of multifactorial traits among identical twins is related with extrinsic aspects, since they nearly share the same genotype (Boomsma et al., 2002).

We tested the hypothesis that having a former world-class champion in the family (ranging from a grandparent to an identical twin) increases the chances of an athlete to repeat the achievement of her/his kinship. We also hypothesized that this probability rises with the degree of relatedness. We therefore aimed to measure the heritability, i.e., the transmission of a trait from parent to progeny – of an Olympic medal and the percentage of the genetic contribution to such a heritance through a twin-family study.

## Materials and Methods

### Study Population and Design

To measure high sports’ performance heritability, we transposed the classic models of disease heritability to the sports field, in which athletic performance is the studied trait. However, contrary to diseases, being an elite athlete is not a trait that you suffer from; it is, instead, a behavior-modulated pursued goal (Pérusse et al., 2001; Güllich and Emrich, 2014). Hence, two peculiarities result when applying classical models of heritability to the study of sports performance traits: (1) individuals may have former elite athletes in their family and inherit their traits, but they may also not be interested in becoming athletes, or did not have the opportunity to do so. Without willingness and directed training, traits expressed in sports performance cannot be revealed; (Ericsson et al., 1993; Baker and Horton, 2004) (2) the second particular feature is related to high-level athletes from families that have not been involved in sports. It is not possible to determine, *a posteriori*, if the kinships of the first athlete in the family would have been world-class champions or not. To avoid bias from the first peculiarity we focused on a population already engaged in high-level sports, including only individuals willing to compete. To overcome the second peculiarity, we focused our analysis on elite athletes who have already undergone a sport-selective filter in order to balance the predisposed conditions at the study’s entry. Hence, we studied athletes who participated in the Olympic Games (OG). The selectivity of the Olympic filter allows access only to the best competitors of each country, selecting athletes who gather both favored genetic and environmental factors to perform at a high level (Antero-Jacquemin et al., 2014; Berthelot et al., 2015). This first threshold is intended to homogenize the conditions of entry in the study, in terms of willingness, training, health and predisposition. Therefore, all women and men participating in the summer or winter OG at least once between 1896 and 2012 were included in the study.

### Data Collection

We collected biographic and performance data for all Olympic athletes participating in the summer and winter Games from 1896 to 2012: Olympians’ full name, date of birth, gender, year of participation(s), sport, medal(s) obtained and kinship(s) also being an Olympian. Olympians kinship data came from a reliable historians’ database (Clarke et al., 2012) that draws on multiple sources (e.g., official competitors lists, birth registers, newspapers, published obituaries) available online^1^. We systematically verified and confirmed these data with data from the International Olympic Committee (Olympic.org) and data from each official website of International Federations archiving athletes’ biographies. Information regarding twins, whether dizygotic (DZ) or monozygotic (MZ), was part of the historical data. In order to confirm their zygosity, we collected the following Supplementary Information: height, public report or self-report in relation to their zygosity, and description regarding concordance in their appearance, and finally their picture. Twins differing in gender, height, or publicly reported to be a fraternal twin, or data profile or picture displaying phenotypic differences, were considered fraternal twins. The phenotypic analysis and self-reported description have been shown to be a reliable method in determining twin populations (Kasriel and Eaves, 1976; Heath et al., 2003). For this type of study, formal consent is not required as all calculations use publicly available data.

### Data Analyses

#### Probability of Medal

Assuming that minimal predispositions to sports are leveled off at baseline among Olympians, a second threshold was chosen in order to establish a higher level of performance and identify its heritability: the Olympic medal; which defines a concrete level of performance expression and is a common pursued goal between elite athletes. A medallist was defined as an individual that has reached the Olympic podium at least once. Then, there were only two possible status, medallist or non-medallist.

The expected probability to win a medal in the Olympics was defined as the frequency of medallists among Olympians without any blood kinship in the OG. Then, this expected probability was compared with the probability to win a medal in the following two groups: Group 1, Olympians with a former non-medallist kinship in the OG and Group 2, Olympians with a former medallist kinship.

The probability of winning a medal in Groups 1 and 2 was calculated for each of the following kinships: between grandparents-grandchildren, aunt/uncle-niece/nephew (the coefficient of relatedness is known to be 25% on average), parents-offspring, full siblings and DZ twins (with a 50% coefficient of relatedness), and monozygotic twins (MZ: 100%).

The probability of a medal in Group 2 was also calculated in two subgroups depending on whether the Olympians were engaged, or not, in the same sport as their medallist kinship.

#### Twin Study

The probability of a medal was calculated among all pairs of DZ and MZ twins with at least one Olympic medallist co-twin. The pairs of twins engaged in collective sports were excluded since the collectivism may smooth a possible relevant difference between twins’ levels of performance.

#### Participation Gap

The number of years separating the first OG participation of an Olympian from their former kinship was measured in Group 2.

#### Historical Trend

Taking into account the possible variations in the prevalence of medallists along the OG history, we compared the probability to win a medal among Olympians having a former sibling medallist in the OG to Olympians without any kinship, according to four historical periods: before World War I, 1896–1912; between World Wars, 1920–1936; before the great boycotts 1948–1976, and the late period 1980–2012 (Guttmann, 2002). Siblings were chosen since they are the most prevalent kinship in addition to having a shorter participation gap.

### Statistical Analysis

The chi-square test was performed in order to test the hypothesis whether the performance level (medallist or non-medallist) is independent among the studied groups and to compare it with the expected frequency of medallists, at a 0.05 significance level. Confidence intervals for the frequencies calculated were stated at 95%.

To bring results among twins comparable to other kinship groups, concordance was estimated by probandwise concordance rates, which are analogous to estimates of recurrence risk in other groups of relatives (Kyvik et al., 1995). Let C_i_ denote the number of concordant pairs in the group, where both twins were medallists, and D_i_ the number of discordant pairs for each group of zygosity. The proband concordance rate is denoted by 2C_i_/(2C_i_ + D_i_). The chi-square test was performed to test the difference of concordance between DZ and MZ based on the pairwise rates, which is the proportion of concordance among all pairs affected.

The heritability (h^2^) variation of the genetically determined phenotype was estimated according to classical methods from twice the difference between MZ and DZ concordance (Boomsma et al., 2002).

Student’s *t*-test was performed to test differences of participation gaps between kinship groups. R software version 3.2.3 was used for all analysis.

## Results

A total of 125,051 athletes participated in the Olympics from 1896 to 2012; 5,661 (4.5%) athletes were related with other Olympians, from which 1,404 (1.1%) had more than one kinship in the Games. For the latter, we considered only the kinship with the greater coefficient of relatedness. Among Olympians without any blood kinship in the Games (*n* = 119,390) 24,319 were medallists. Therefore, the expected historical probability to obtain a medal was 20.4%.

### Group 1) Kinship With Former Non-medallists Olympians

**FIGURE 1:**
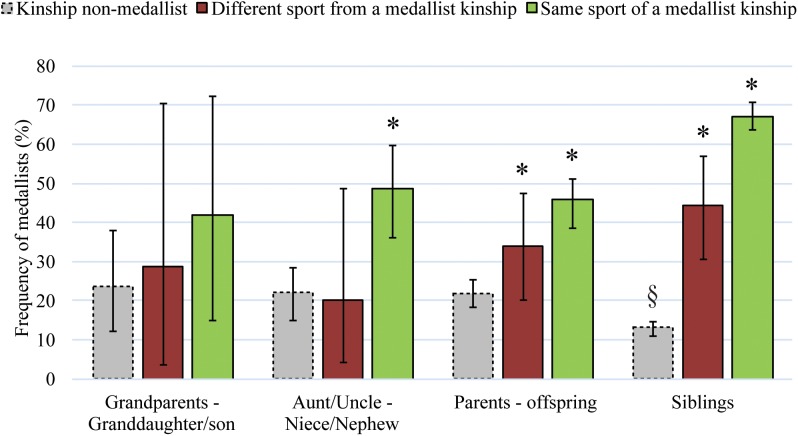
Frequencies (%) (± 95% CI) of Medallists having a grandparent, aunt/uncle, parent or siblings in the Olympic Games that were non-medallists (gray bars, dotted line) and that were Medallists (solid bars), in a different sport (red bars) or in the same sport (green bars). ^∗§^ Frequency of Medallists significantly different (*p* < 0.001) in relation to the expected probability (20.37%).

Considering athletes with a kinship with another Olympian, 38 had a grandparent, 144 had an aunt/uncle, 564 had a parent and 1,364 had an older sibling in the games, who were non-medallists. The frequency of Medallists in these groups (**Figure 1**) was not significantly different from the expected probability, except for Olympians who had a non-medallist sibling, among them the frequency of Medallists found was lower, 13.3% (95% CI 11.2–14.8).

### Group 2) Kinship With Former Olympic Medallists

The probability to obtain a medal in the Olympics according to a kinship with a former Olympic Medallist is detailed in **Table 1**.

**Table 1:** Frequency of medallists according to a kinship status with a former Olympic Medallist.

Kinship	Coefficient of relatedness (on average)	Number of Olympians (*n*)	Medallists (*n*)	Frequency of medallists in the subgroup (95% CI)	Chi-square test^∗^	Participation gap years Mean (± SD)
Grandchildren	25%	19	7	36.84% (20.2–59.4)	*p* > 0.05	55.9^a^ (± 12.3)
Niece/nephew	25%	90	40	44.44% (33.7–54.2)	*p* < 0.001	20.6^b^ (± 13.0)
Offspring	50%	295	128	43.38% (37.3–48.6)	*p* < 0.001	29.3 (± 10.4)
Full siblings	50%	608	394	64.80% (61.2–68.8)	*p* < 0.001	6.7 (± 3.8)
DZ twins	50%	53	40	75.47% (63.3–86.6)	*p* < 0.001	1.0^c^ (± 2.0)
MZ twins	100%	21	18	85.71% (63.7–96.9)	*p* < 0.001	1.0 (± 1.8)

*^∗^Test the hypothesis whether there is a significant difference between the expected frequencies of medallists according the group of Olympians with no kinship and the observed frequencies in the groups of Olympians with former Medallists kinships. ^*a*^Participation gap is significantly greater than among niece/nephews (p < 0.001) and offspring (p < 0.001). ^*b*^Participation gap is significantly shorter than among offspring (p < 0.001) and greater than among siblings (p < 0.001). ^*c*^Participation gap is significantly shorter than among siblings (p < 0.001) without difference with MZ twins (p > 0.05).*

#### Grandchildren

The probability of winning a medal among grandchildren of former Olympic Medallists was not significantly different from the expected probability (**Table 1**), whether for those engaged in the same sport as their grandparents – a slight majority (52.83%) – or not (**Figure 1**). The participation gap between these generations was the largest (*p* < 0.001) within the kinship groups studied (**Table 1**).

#### Niece/Nephew

The probability of a medal among nieces and nephews of Olympic Medallists was greater and significantly different from the expected probability (**Table 1**), led by the majority of nieces and nephews (78.16%) that have competed in the same sport as their kinship (**Figure 1**). For the athletes engaged in a different sport as their aunt/uncle the probability of a medal was equivalent (*p* > 0.05) to Olympians with no kinship (**Figure 1**).

#### Offspring

The probability of winning a medal among daughters and sons of Olympic Medallists was greater and significantly different from the expected probability (**Table 1**). Whether for the majority of athletes (80.72%) that have competed in the same sport as their parents as well as those competing in a different sport (**Figure 1**). The participation gap between parents-offspring was significantly greater than the pairs of aunt/uncle-niece/nephews (*p* < 0.001) (**Table 1**).

#### Full Siblings

The probability of a medal among sisters or brothers of former Olympic Medallists was greater and significantly different from the expected probability (**Table 1**). Whether for the vast majority of athletes (92.37%) engaged in the same sport as their older siblings or for those competing in a different sport (**Figure 1**). The participation gap between siblings was significantly shorter than that found between the pairs of aunt/uncle-niece/nephews, or parents-offspring, and significantly greater than that found among twins (**Table 1**).

#### Twin Study

**FIGURE 2:**
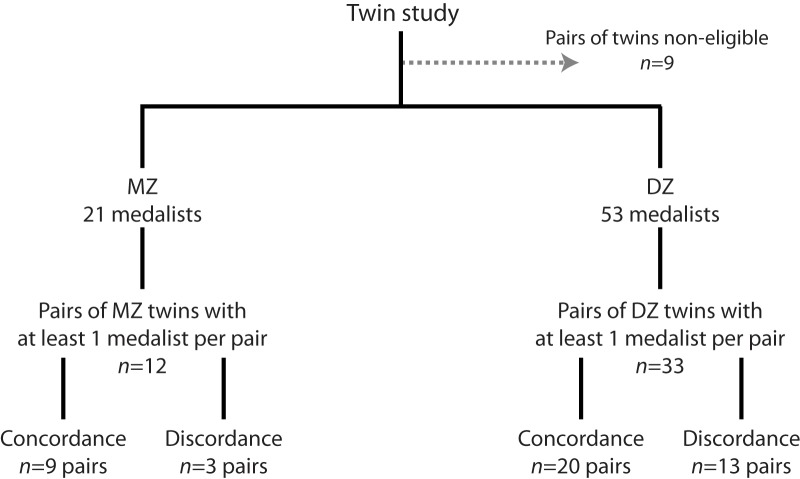
Twin study flow chart.

All pairs of twins, among whom at least one of them was a Medallist, were identified according to their zygosity: 33 were DZ twins and 12 were MZ. Among them, 20 pairs of DZ twins and 9 pairs of MZ twins were concordant for the medal trait (**Figure 2**).

The probability to win a medal in the OG, given a Medallist DZ or MZ co-twin was greater and significantly different from the expected probability (**Table 1**). Significantly greater medal concordance was found between MZ than DZ (*p* = 0.01), with no significant difference between both kinship groups regarding the participation gap (*p* > 0.05). The heritability variation of the genetically-determined phenotype was estimated at h^2^ = 20.48% for winning a medal after entering the OG.

**FIGURE 3:**
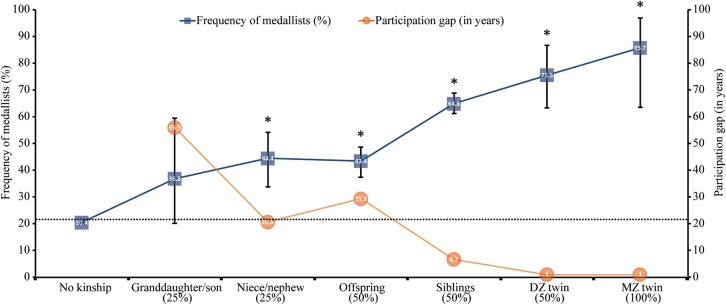
Frequencies (%) (± 95% CI) of Olympic Medallists (blue squares) according to an average gradient of relatedness (x-axis labels %) with former Medallists in the Olympic Games. Participation gap (orange circles) represents the mean of years separating the first participation in the Olympics between each pair of kinship. The dotted vertical line shows the average prevalence of medals in the history of the Olympic Games. ^∗^The Medallist frequency is significantly different (*p* < 0.001) from the expected one.

The frequency of Medallists in Group 2 is illustrated in **Figure 3** following a gradient of relatedness relying the kinship/no kinship groups studied and the years of participation gap between the pairs of kinships.

### Historical Trend

**FIGURE 4:**
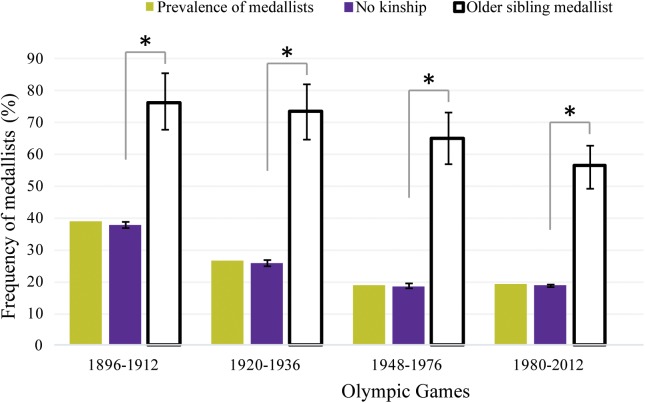
Prevalence of Medallists in the Olympic Games according to four historical periods (yellow bars). The purple bars represent the frequency of Medallists (%) (±95% CI) among Olympians with no kinship in the Games per period. The white bars represent the observed frequency of Medallists (%) (±95% CI) among Olympians with a former sibling Medallist in the Olympics for each period. ^∗^The Medallist frequency was significantly different (*p* < 0.001) from the expected one.

The prevalence of Medallists in the OG decreased during the modern Olympic era: from 38.9% in the first period to 19.1% in the last one (**Figure 4**). The probability to be a Medallist among Olympians with no kinship was statistically equivalent to the prevalence related to medal distribution for every period (*p* > 0.05). For Olympians with a former sibling Medallist, the probability was consistently greater (*p* < 0.001) than among Olympians without kinship for each studied period (**Figure 4**). The medal frequencies among Olympians with any kinship was 2.0, 2.7, 3.5, and 3.0 times greater than among Olympians with no kinship for the respective periods: 1896–1912, 1920–1936, 1948–1976, 1980–2012 (**Figure 4**).

## Discussion

### An Olympic Medal Is Heritable

For any participant to the OG, having a kinship with a previous Olympic medallist, is associated with a greater probability to also win a medal. The closer an athlete is genetically to such a kinship and the shorter the Olympic participation gap between them, the greater the probability to take a place on the podium. The likelihood is even greater if both athletes were engaged in the same sport. This trend is present throughout the entire OG era. In the earlier Games, Olympians with former medallist siblings doubled their chance to repeat the achievement of their sister or brother; in more recent competitions, their medal probability is three times greater than for Olympians with no kinship.

Examples are widespread. Consider the family of champions: the Russian-American Olympic champion gymnast Anastasia Liukin, daughter of Valeri Liukin, former Olympic champion, or the French swimmer siblings, Laure and Florent Manaudou, or the Brazilian volleyball players, Bernardinho and Bruno Rezende, father, and son Olympic medallists. All contribute to illustrate this finding: an Olympic medal runs in the family.

The genetic contribution to the heritability of the medal is evidenced by the greater probability to be a medallist when the coefficient of relatedness with an Olympic medallist increases. This probability doubles with a second-degree link to a prior medallist and reaches the very high rate of 85% with medallist MZ co-twins.

Comparing the resemblance between MZ and DZ twins as a trait offers the first estimate of the extent to which genetic variation determines the phenotypic variation of that trait (Boomsma et al., 2002). Both types of twins are expected to share similar environmental conditions from conception, while only MZ are genetically identical. Despite the potentially common environment shared among both DZ and MZ twins (as also suggested by an equivalent participation gap in the Olympics), the probability to be a medallist is greater among MZ twins. Such a difference allows the estimation of the part of the trait that is due to additive genetic factors. In the history of the Games, we estimated the genetic heritability of an Olympic medal to be 20.5% among Olympians.

Predispositions to performance and fitness have been evidenced in various studies, ranging from the general population to elite athletes, and from a specific single nucleotide polymorphism to familial aggregation studies (Wang et al., 2013). This is the first study, however, to measure the heritability of an Olympic medal based on the entire Olympian population.

### Genetic Factors

Performance-enhancing polymorphisms have been associated with specific athletic predispositions related to energy production, metabolic processing, oxygen transport, skeletal muscle formation, blood flow, oxygenation, and muscle structure (Ostrander et al., 2009). Genetic factors account for more than 50% of the inter-individual variation that influences the type and size of the muscle fibers, the innervation type as well as the blood flow (Ostrander et al., 2009), all involved in sports performance. The athletes’ height, which is a common criterion to detect and select elite athletes (Sedeaud et al., 2012), is also highly related to genetic heritance (Visscher, 2008).

Participation in sports has been shown to be inheritable: a child or co-twin is more likely to be active in sports if one of their kinship is also active (Beunen and Thomis, 1999). In adulthood, genes have been argued to largely explain individual differences in sports participation with high rates of heritability (Stubbe et al., 2005). Explosive power, anaerobic capacity (Calvo et al., 2002) and muscle adaptation to strength training are also considered to be heritable (Thomis et al., 1998). Similarly, the heritability of traits, such as maximal heart rate, blood lactate and aerobic performance have been shown in the general population (Lesage et al., 1985; Pérusse et al., 2001). Addressing concordance among siblings, previous studies have shown higher concordance rates of aerobic performance among MZ twins than among DZ twins and other siblings (Bouchard et al., 1986). The HERITAGE study, investigating maximal oxygen uptake (VO_2_max) in sedentary individuals, has estimated maximal heritability at 50% (Bouchard et al., 1998).

Whereas heritability related to performance has been estimated to be moderate to high (Davids and Baker, 2012), previous research has usually compared elite athletes to controls in the general population. In our study the control group is composed of Olympic level athletes. Thus, the genetic heritability estimated for the medallist trait is an additive estimation to the whole set of predispositions that Olympians already demonstrate in order to qualify for the Games. Acceding to the Olympics already requires a first-range of predispositions. This is illustrated by the comparable medal probabilities found between Olympians with no kinship and Olympians with kinship with a non-medallist. Having a kinship in the Games is not an advantage *per se*, supporting the assumption that the selectivity of the Games acts as a first filter balancing the predisposition of the athletes entering the study. After passing the Olympics filter, climbing the podium requires supplementary predispositions (genetic, behavioral and environmental), 20% of which is estimated to be in the athletes’ genes.

The heritability role in the performance level is constant along the Olympic history in view of the findings consistently favoring Olympians with former medallist siblings. The weight of social and environmental factors of a kinship was already quantifiable in the earlier Games. Yet, while the advantage displayed by Olympians with medallist siblings in this period was two times greater than expected, such an advantage still increases in the latest period. Such findings are potentially explained by the greater concurrence and professionalism in the recent competitions (Berthelot et al., 2015), leading to more selective filters and a greater prerequisite to individual predispositions.

### Environmental Factors

Despite the genetic contribution to sport performance, trying to disentangle the part of either nature or nurture on performance heritance is a challenge, as part of the environment is also inheritable. More than genes, relatives usually share common environments and behaviors (Plomin, 2008) creating a socio-cultural heredity that simulates a biological heritance.

The environment favoring athletic performance consists of all extrinsic factors positively influencing the ability to engage in the required amounts of high-quality training (Ericsson et al., 1993; Baker and Horton, 2004). Several resources, usually shared among the members of an athletic family, determine the socio-cultural heritance propitious to performance: training aspects (availability to practice, technicality, training techniques, coaches, medical, and scientific staff) (Vancini et al., 2014), social and financial aspects, (Côté, 1999) career management (Güllich, 2014), family support (Hopwood et al., 2015), and experience exchange (Davids and Baker, 2012; Tucker and Collins, 2012).

The socio-cultural contribution to performance is pictured here through the Olympic participation gap, which represents the environment alikeness shared between relatives and the correspondence of sports. Accordingly, the agreement of sports between the pair of kinships is greater when the participation gap is shorter. This socio-cultural heritability may be more easily transmitted through a shorter participation gap.

The similar frequency of medallists in the pairs of kinship aunt/uncle-niece/nephew and parents-offspring, despite the difference in relatedness between these kinships, may illustrate the importance of the socio-cultural heritability. Nieces and nephews have a significantly shorter Olympic participation gap than offspring in relation to their parents. The 10 years difference may explain the similar probabilities of medallists found in these groups. Likewise, kinship pairs of parents-offspring, siblings and DZ share the same degree of relatedness, but the probability of a medal is greater as the participation gap is shorter according to a non-linear relation.

These findings suggest that the socio-cultural heritance may also play an important role in the development of elite athletes’ careers. Family members have been shown to be highly influential in the development of sport expertise (Hopwood et al., 2015). The commitment to intense athletic training has been suggested to be the result of common genes and shared environment (Plomin, 2008). A closer environment shared with a former champion may enhance the genetic predisposition, while not experiencing common resources in sports may weaken the genetic propensity. In addition, a kinship with a champion may place a child in the spotlight as a potential talent, leading to an early onset of training and access to facilities, procuring an environmental advantage in addition to the genetic predisposition. This intricate relation between biological and socio-cultural heritance strengthens the complex interplay between genetics and environment on elite performance (Davids and Baker, 2012; Marck et al., 2017).

### Sport Performance Is a Multifactorial Trait

High performance in sports is consistent with a combination of distinctive characteristics allowing its classification as a multifactorial trait: numerous factors concur to the trait onset. Greater concordance is observed among identical twins than in DZ twins; the trait frequency increases with the degree of relatedness; it runs in families without Mendelian heritance patterns; it can occur in isolation (a kinship is not necessary for the trait), and environmental influences modulate the chance of the trait onset (Boomsma et al., 2002; Yusuf et al., 2004; Watkins and Farrall, 2006).

In a multifactorial trait, the susceptibility to a given phenotype is distributed in the population following a bell-shaped curve (Watkins and Farrall, 2006; Plomin et al., 2009). The sports performance trait exhibits a Galtonian pattern following a continuous variation, i.e., gradation in expression with no abrupt change from one phenotype to another. The susceptibility being additive, most individuals have an intermediate phenotype (Plomin et al., 2009). The extremities of the curve represent individuals cumulating completely favorable or unfavorable conditions, therefore representing rarer phenotypes, the farther from the mean they are (Plomin et al., 2014; Marck et al., 2017). Olympic athletes reach the right extremity of the bell-shaped curve of performance, the Olympic medallists being the most right shifted.

The model of quantitative gradient of susceptibility in sports helps to explain the present findings. The likelihood of reaching the podium is greater among the most right-shifted athletes in relation to the Olympians’ bell-shaped curve: such is the case of those with a medallist kinship. This model also allows for understanding the reason of consistently lower medal frequency when engaging in a different sport than that of a former medallist kinship. By choosing a different sport, the succeeding kinship partially reduces both genetic and environmental predispositions. The former medallist kinship has probably gathered a great amount of rare favorable conditions allowing her/his achievement. As each sport demands specific capacities, changing sports requires new capacities. The probability for Olympians to accumulate both the conditions inherited from the kinship and the newly required ones are lower. The same rationale may explain the lower probability found among Olympians with a non-medallist sibling. The medal predisposition among siblings with a sister/brother medallist is stronger than among the kinships of grandparents–grandchildren, aunt/uncle-niece/nephew and parents–offspring. Kinship with non-medallist sibling suggests a strong relation with someone predisposed to high level performances, yet, probably missing the supplementary 20% set of genetic predispositions needed to reach the Olympic podium. Considering elite athletes as super-controls in quantitative traits of performance may have far-reaching implications in apprehending the extremes of such traits in case control studies (Plomin et al., 2009).

### Limitations and Strengths of the Study

The zygosity established according to the phenotype, although reported to be reliable, is susceptible to misclassification. In addition, our data restrains the twin study to a classical approach, which cannot accommodate the effect of covariates on variances (Boomsma et al., 2002), and does not include a component for interaction of genotype and environment (Davids and Baker, 2012). Hence, a more specific analysis including age at peak performance or ethnic heritability could not be performed, despite the importance of such parameters in understanding sports performance heritability. An integrationist design of sports performance combining genomics, epigenomics, and transcriptomics (Bouchard, 2011; Hoffman, 2017) using more sophisticated quantitative models may provide a better understanding in the determination of athletic potential, which seems to impact lifespan (Antero-Jacquemin et al., 2015; Marck et al., 2017).

Our methods’ advantages, however, rely on the analyses of the entire population of Olympians, including the analyses of all kinships among the highest-level athletes, and thus providing a complete heritability description of this population, as previously recommended (Wang et al., 2013).

## Conclusion

Nieces/nephews, offspring, siblings, and twins of former Olympic medallists have a greater probability to win a medal in the OG than competitors with no kinships. The closer the genetic relation to a former medallist and the shorter the temporal gap between their Olympic participation, the greater the probability to win a medal, especially if they participate in the same sport. The advantage of having a kinship medallist is constantly observed over a century of Olympic history. The genetic contribution to the heritability of an Olympic medal is estimated to be 20.5% among Olympic athletes. A wide range of intricate elements work together at a bio-environmental level to define the optimal and measurable physical performance.

## Author Contributions

JA and J-FT came up with the study idea. JA collected and analyzed the data with assistance from GS and AM. All authors contributed to writing the article and participated in the scientific debate. J-FT is the guarantor.

## Conflict of Interest Statement

The authors declare that the research was conducted in the absence of any commercial or financial relationships that could be construed as a potential conflict of interest.

## Footnotes

1 www.sports-reference.com/olympics
